# Effects of macroprudential policies on ecological footprint: the moderating role of environmental policy stringency in the top 11 largest countries

**DOI:** 10.1038/s41598-024-58015-9

**Published:** 2024-03-28

**Authors:** Heng Luo, Ying Sun, Li Zhang

**Affiliations:** 1 School of Digital Economy and Industry, Jiangxi University of Engineering, Xinyu, Jiangxi China; 2 School of Business & Economics, Universiti Putra Malaysia, Serdang, Malaysia; 3 School of Economics, Zhongnan University of Economics and Law, Wuhan, Hubei China

**Keywords:** Macroprudential policies, Ecological footprint, Environmental policy stringency, Quantile regression, Top 11 largest countries, Environmental social sciences, Environmental economics, Environmental impact

## Abstract

This study investigates the impact of macroprudential policies on ecological footprint (EF) in the top 11 largest countries. This study uses country-level panel data from these countries, covering the period from 1992 to 2020. Findings indicate that macroprudential policies alleviates ecological footprint in the sample. Macroprudential policies primarily reduce the ecological footprint before medium quantile (50%) while the environmental benefits of the policies end in the later quantiles. Moreover, environmental policy stringency (EPS) amplifies the positive influence of macroprudential policies on environmental sustainability. Estimate results stay the same with basic regression results in the post-global financial crisis (GFC) period while the impact is positive in the pre-GFC period. Finally, other robust tests validate the findings reported in basic regression model. This study suggests that governments should customize various types of macroprudential policies while also considering environmental concerns. The achievement of a sustainable environment can be facilitated by the combined effects of macroprudential policies and EPS.

## Introduction

**Figure 1:**
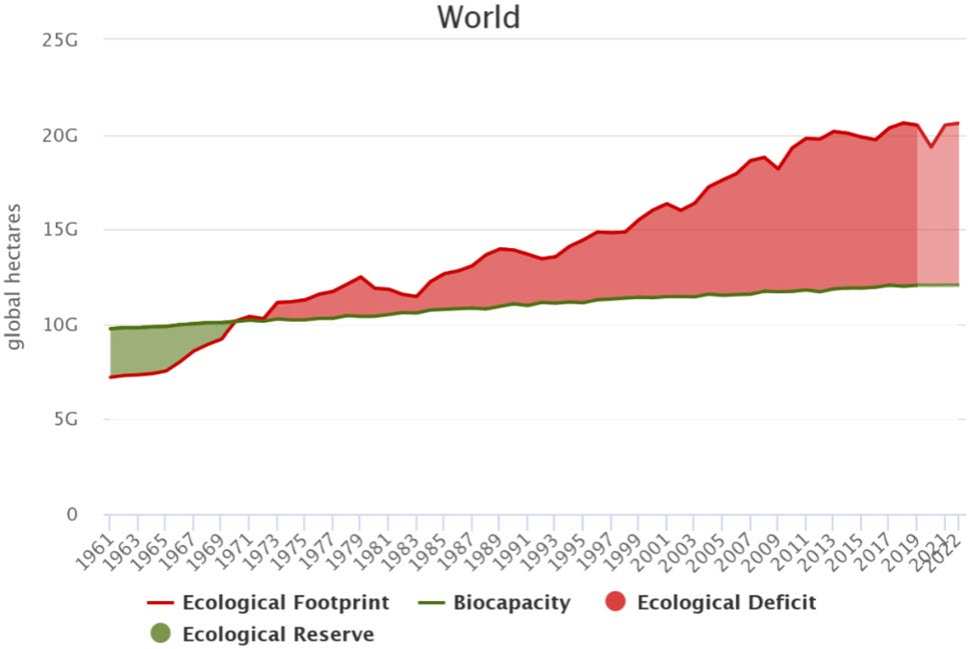
Gap between ecological footprint and biological capacity for over 200 countries and regions from 1961, source from Global Footprint Network.

The escalation of industrialization has brought environmental issues to the forefront of scholarly and political discussions. It has been identified as a critical concern that must be addressed in order to achieve sustainable economic growth. The United Nations Framework Convention on Climate Change (UNFCCC) has reached a consensus that greenhouse gas (GHG) emissions have a significant impact on global warming. Industrialized countries bear a greater responsibility in addressing this issue^1^. The Kyoto Protocol outlined the objectives and procedures required to carry out UNFCCC. According to this protocol, Between 2008 and 2012, the 40 most industrialized nations must lower their emissions by at least 5% from their 1990 levels^2^. The Paris Agreement was adopted under the UNFCCC in 2015, aiming to restrict temperature to below 2 °C, preferably 1.5 °C^3^. The results of COP 27 show that it had less success tackling the effects of climate change and stress the importance of holding the line on 1.5^4^. Previous studies^5,6^ use CO2 emissions to evaluate the extent of environmental contamination in the air. However, it’s important to consider that other factors occurring in lands and grasslands also significantly influence the environment. A more inclusive indicator known as the ecological footprint developed by^7^ is more appropriate for measuring environmental issues. It is considered more precise than focusing solely on CO2 emissions^8^. The number of resources that humans demand from nature and the capacity of nature to supply those resources are measured by the ecological footprint. The demand part gauges the ecological resources needed by a particular population to produce the natural resources it consumes as well as absorb its waste. From the supply part, biocapacity measures how productive its ecological resources are. If a region’s ecological footprint deducts its biocapacity is over 0, then the region experiences a biocapacity deficit. Otherwise, it has a biocapacity reserve. According to the data from Global Footprint Network (GFN), as depicted in Fig. 1, the world experienced a biocapacity reserve before 1970, while in later years it was in a biocapacity deficit. By 2018, the ecological footprint had reached 1.5 times the biocapacity, surpassing the limit considered sustainable for the future^9^.

To promote sustainability and preserve the planet’s health for future generations, people must reduce ecological footprint. As a result, the fundamental issue faced by policymakers is how to sustain environmental health while taking into account economic growth. Previous scholars have explored many factors that will alleviate biocapacity deficit, such as financial development^10,11^, foreign direct investment^12,13^, EPS^14,15^, and renewable energy consumption^16,17^. However, the macroprudential policies-environmental degradation nexus has hardly been explored. As defined by IMF (https://www.imf.org/external/np/g20/pdf/2016/083116.pdf), macroprudential policies are “the use of primarily prudential tools to limit systemic risk”. The literature on this nexus is in its infancy. Additionally, prior studies on the connections between macroprudential policies and environmental degradation have yielded contradictory results. Some scholars argue that macroprudential policies appear to value quick investments in the environment over “patient” (long-term) green ones^18–20^, which will depress environmentally friendly investments. Then these policies will increase environmental degradation. In contrast, by allocating lower reserve requirements for green loans, green credit will expand^18^. In the meantime, a higher countercyclical capital buffer is required for carbon-intensive credit. Reduced carbon-intensive credit and increased green credit will help reduce environmental problems.

Against this background, this study aims to empirically examine the nexus between macroprudential policies on the ecological footprint in the top 11 largest countries. There are three factors that influence the sample selection. First, these top eleven countries by GDP were chosen for this research based on World Bank statistics for 2019. These countries include Brazil (2.14%), Russia (1.93%), India (3.23%), China (16.28%), Canada (1.99%), France (3.11%), Germany (4.43%), Italy (2.29%), Japan (5.83%), the United Kingdom (3.26%), and the United States (24.37%). Despite comprising the world’s leading economies and accounting for up to 68.86% of global GDP (current US$), these countries face environmental challenges just like any other nation. Second, according to the data from the Environmental Protection Agency, these countries are among the top 25 nations with the highest total GHG emissions in 2020, with the exception of the United Kingdom, which ranks 31st, and Italy, which ranks 37th. Additionally, ecological footprint measures the demand and supply of nature, making it an indicator of environmental status. According to the data from the Global Footprint Network, all these 11 countries remain among the top 15 countries with the largest ecological footprints (gha) worldwide in 2022. Except for Russia, Brazil, and Canada run an ecological reserve, all these countries experience an ecological deficit, which signifies that their ecosystems cannot sustain the demand for the commodities and services that their land and oceans can offer. Third, these nations are signatories to both the Paris Agreement and the Kyoto Protocol. They have vowed to promote sustainability and solve the world's environmental problems, including climate change, and to lessen or eliminate greenhouse gas emissions.

This study differs from earlier research in the following parts. First, to the best of our knowledge, this is the first paper to examine the impact of macroprudential policies on ecological footprint. Second, despite a wealth of studies published in the literature examining the relationship between EPS and environmental problems, considering EPS as a moderating variable in the context of the environment remains an underexplored research area. In this context, the study will contribute to the literature on environmental issues and ensure the overall findings for measures that governments formulate to alleviate strain on the environment. Third, this study uses quantile regression to analyze the non-liner effect of macroprudential policies on ecological footprint. This approach shows the different effects of macroprudential policies across the conditional distribution of ecological footprint, which helps it overcome the drawback of traditional methods that focus on the conditional mean.

In the regression model, macroprudential policies are the independent variable, while the dependent variable is ecological footprint, a more comprehensive index of environmental damage called ecological footprint, which measures how much area of biologically productive resources the population requires. Additionally, EPS is the moderating variable. Regression results indicate that macroprudential policies reduce ecological footprint. Quantile regression results indicate macroprudential policies primarily reduce the ecological footprint before the medium quantile (50%) while increasing the ecological footprint in the later quantiles. EPS strengthens the positive role of macroprudential policies in promoting sustainability. To promote sustainability and mitigate environmental damage, countries are advised to integrate macroprudential policies with considerations of economic growth, while also coordinating efforts with EPS.

The remaining section of the paper is arranged as follows: section “Literature review” is the literature review. Section “Data and methodology” describes the data and methodology. Section “Empirical results” reports the empirical results. The last section emphasizes the key conclusions.

## Literature review

### Macroprudential policies and ecological footprint

**Figure 2:**
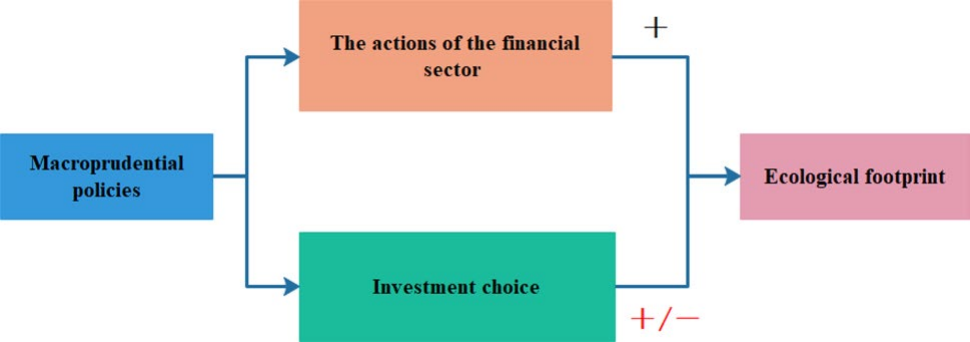
The mechanism of macroprudential policy on EF.

Despite the fact that research on the environmental effects of macroprudential policies is still in its early stages, two mechanisms depicted in Fig. 2 may explain the impact of macroprudential policy on EF.

In terms of the former, macroprudential policies influence the actions of the financial sectors, then influence EF. For example, these policies categorize loans that are allocated to practices that increase ecological footprint as “excessive”, whereas loans that contribute to reducing the ecological footprint as “scarce”. Countercyclical capital buffers would be higher when financial institutions offer more credit to carbon-intensive firms^21^. A larger buffer requirement essentially means that financial institutions are required to hold more capital as a proportion of their total assets, which will reduce the profit of institutions. In addition, capital instruments can allocate more risk weight to assets that increase EF, while assigning less weight to assets that reduce the EF^22^. This approach would integrate the added 'carbon risk' into the overall risk-return assessment within the financial sector’ action. Finally, when confronting green loans, differentiated reserve requirements have resulted in the allocation of less reserves^23^. Because of this, financial sectors are able to lend out more money, which boosts their volume of business and encourages them to extend green credit.

In terms of the latter, these policies influence investment choices, which in turn affect EF. On the one hand, macroprudential policies promotes green investments, thereby reducing EF. Financial stability can be effectively promoted by macroprudential policies^24^. Financial stability can lead to more predictable market conditions, which in turn can facilitate long-term planning and investments in sustainable practices that can reduce EF. This view is consistent with the research of^25^, who conclude that financial stability is beneficial for reducing EF. On the other hand, policies implemented in the wake of the financial crisis, most notably Basel III, seem to encourage “immediate” (i.e., short-term) over “patient” (i.e., long-term, green) ones. This view aligns with the research conducted by^19^ as well as^20^. The decrease in green investment has significant environmental repercussions, especially in terms of how it would affect the ecological footprint. When green investments fall, funding for environmentally friendly and sustainable projects also declines. Therefore, there may be an increase in ecological footprint if natural resource demands outpace their capacity for regeneration. However, there is also evidence in the literature of a beneficial association between macroprudential measures and green investment. Maximum credit ceilings and minimum credit floors offer a very straightforward mechanism for directing investments toward “green” projects^26^. While the latter necessitates institutions to allocate a minimum amount of resources to green investment, the former operates by imposing limits on the maximum bank exposures to carbon-intensive or polluting industries. In the work of^27^, the brown penalizing factor assigns higher risk weights to polluting investments, whereas the green supporting factor functions by assigning lower risk weights to environmentally friendly projects. As a result, environmentally friendly activities increase while polluting ones decrease due to the different preferences of financial institutions.

### Environmental policy stringency and ecological footprint

Governments have implemented laws in response to rising environmental consciousness in order to reduce the cost of environmentally harmful behavior. The EPS developed by the Organisation for Economic Co-operation and Development (OECD) measures the level of stringency defined as the expense of contamination. According to^28^, a well-designed policy can assist firms in implementing eco-friendly technologies, which can result in a reduction in pollution. Therefore, by promoting the deployment of clean technologies, EPS can mitigate environmental damage^14^. Similarly, EPS is thought to have the ability to lessen pollution's negative impacts by encouraging the development of “clean” technology and discouraging the use of “dirty” ones^29^. Additionally, by making it more expensive to produce “dirty” items to the point that they become unattractive, EPS works to encourage sustainability^30^. However, it’s important to consider the EPS-related costs such as postponing investments in green innovative technologies^31^ and certain kinds” of innovation^32^. These costs may deepen pollution. In terms of empirical evidence, the research concerning the connection between ecological footprint and EPS is still in its infancy. In the case of OECD countries, EPS is documented to alleviate the ecological footprint^14,15^. In the case of APEC countries, a similar conclusion is drawn by^33^. When other works evaluates environmental deterioration using various variables, the conclusions of researchers are full of controversy^34^. found EPS is effective in reducing CO2 emissions for 20 European countries^35^, conclude that the improvement in environmental quality is due to EPS for BRICST countries^36^, found the emission level is negatively connected with EPS for 32 OECD countries. However, the “green paradox”^37^ also exists. This theory shows that EPS could have unintended, undesirable impacts that accelerate environmental pollution. According to GMM findings of^38^, EPS have not been as effective in controlling and reducing pollution as they were supposed to be. EPS is to blame for the rise in carbon emissions in Asia^39^.

Furthermore^33^, explore the moderating effect of EPS on the nexus between financial development and ecological footprint and conclude that EPS amplifies the positive impact of financial development on pollution lessening in APEC countries. A similar result is found in the work of^40^, who conclude that EPS amplifies the positive impact of natural resources rent on green growth.

In light of the complex and inclusive conclusions, it is important to investigate whether the impact of macroprudential policies on environmental sustainability in the top 11 largest countries can be moderated by the EPS.

## Data and methodology

To analyze the influence of macroprudential policies on environmental sustainability in the top 11 largest countries. This study gathers data from a variety of sources. Firstly, ecological footprint is selected as the indicator for environmental sustainability from https://data.footprintnetwork.org/#/. Secondly, the MaPR_3 index is utilized as a measure of the level of macroprudential policies, and for this purpose, the relevant data is acquired from^41^, who have built a database about macroprudential policies based on annual records. Thirdly, the country-level control variables are gathered from WDI. Finally, this study opts to use the EPS Index from the OECD database, serving as the moderating variable. The panel data of these countries were taken from 1992 to 2020 to integrate the variable’s associations. Since the MaPR_3 index covers the period from 1992 to 2021, while the OECD database includes EPS Index data for the years 1990 to 2020, the sample period for this analysis is from 1992 to 2020.

### Dependent variable: ecological footprint (EF)

Adopting the approach used by^42,43^, this study utilizes ecological footprint constant per capita(log.) (https://data.footprintnetwork.org/#/) as dependent variable.

### Independent variable: macroprudential policies

The implementation of macroprudential tools can be traced back to before the global financial crisis. They play an essential role in managing credit, directing investment, and maintaining financial stability. In order to get a general indicator of macroprudential policies that aggregates the usage of multiple macroprudential tools, this study collect macroprudential tools ‘data from^41^, who collect the history of a wide range of macroprudential instruments including 17 distinct measures, on a monthly basis for the period spanning from 1990 to 2021. Each macroprudential measure was given a numerical value by the researcher, with 1 denoting a tightening tool, − 1 denoting a loosening tool, and 0 denoting otherwise. The sum of the numerical values attributed to the 17 macroprudential indicators yields the aggregate index for each nation for a given month. For instance, if one country loosens the requirements for Loan loss provision and Limit on leverage of banks, tightens Limits to the loan-to-value ratios, and maintains neutrality for the other macroprudential instruments in a given month, then the aggregate index for that country in that month will be – 1 − 1 + 1 = − 1. The value of macroprudential policies is a net value, representing the totality of the country’s tightening and loosening tools.

Although monthly access to the aggregate index is available, the study’s variables must be annual. To create yearly variables, the researcher aggregates the index values for every month within a given year. The resulting sum represents the macroprudential policy intensity for that specific year. A policy intensity that is tighten-oriented for that year is indicated by a positive value, whereas a policy position that is loosen-oriented is shown by a negative value. Using this annual aggregate, the study may look at longer-term trends and changes in macroprudential policy.

It is unpredictable when these macroprudential regulations will impose an effect on banks and borrowers^44^. Similarly, the implementation of macroprudential actions, such as changes in capital requirements, loan-to-value ratios, or reserve requirements, can have a delayed effect on the environment. The time it takes for the effects of macroprudential policies to manifest on the ecological footprint is referred to as the transmission lag. Additionally, macroprudential regulations often continue to have an impact for years after their initial implementation. Due to these factors, solely focusing on the current influence of macroprudential policies may obscure their long-term effects on the ecological footprint. To assess the value of macroprudential policies for a specific year, this study adopt the approach of^45,46^ by using a three-year aggregated value to represent the intensity of macroprudential policies in that year. The true value of macroprudential regulation in year t is replaced with MaPP_3. For instance, the value for country i in 1990, 1991, and 1992 is 1, 2, and 3 individually. Consequently, the MaPP_3 employed for 1992 is calculated as (1 + 2 + 3) = 6. The MaPP_3's time range is from 1992 to 2021 because the database spans the years 1990 to 2021, however this study produces MaPP_3 using three-year aggregated values.

### Control variable

Previous research indicates that the external macroeconomic environment has an impact on ecological footprint. With reference to^33,42,43,47–49,49^, this study choose the following variables: (1) fiscal policies: general government final consumption expenditure (% of GDP) is used to characterize it. (2) Economic growth(GDP): GDP per capita (constant 2015 US$)(log) is selected as the proxy of economic growth. (3) Openness index(trade) is measured by the ratio of the sum of total exports and total imports to GDP. (4) fdi is proxied by foreign direct investment, and net inflows (% of GDP), (5) financial development (FD) is the share of the domestic credit to the private sector of the GDP.

### Moderating variable

The demand for instruments to compare nations' environmental policy stringency is growing as countries implement more stringent environmental rules. This study uses the EPS index from the OECD environmental statistic database which combines quantitative and qualitative data about environmental policy. This database compiles data on selected different environmental policy tools, mostly those that deal with climate change and air pollution. This index allows for a measurable assessment of the extent to which environmentally harmful activities are affected by environmental policies. A lower value denotes a less strict policy, with zero signifying lax regulations. This study follows the method of^33,40^, taking environmental policy stringency as the moderating variable.

Data for the variables above are summarized in Table 1.

**Table 1:** Variables explanations.

	Variable	Symbol	Description	Source
Dependent variable	Ecological footprint	ef	Ecological footprint constant per capita(log.)	https://data.footprintnetwork.org/#/
Independent variable	Macroprudential policies	MaPP_3	Measure the level of macroprudential policies	(Alam et al.^41^) (https://www.elibrary-areaer.imf.org/Macroprudential/Pages/Home.aspx)
Control variables	Fiscal Policy	FP	General government final consumption expenditure (% of GDP)	WDI
	Economic growth	GDP	GDP per capita (constant 2015 US$)(log)	WDI
	Openness index	trade	Total exports + total imports (% of GDP)	WDI
	Foreign direct investment	fdi	Foreign direct investment, net inflows (% of GDP)	WDI
	Financial development	FD	Domestic credit to private sector % of GDP	WDI
Moderating variable	Environmental policy stringency	EPS	The proxy of environmental law	OECD environmental statistic database

### Econometric model

This step of this study was to find out the impact of macroprudential policies on ecological footprint by MPRA, which included the ordinary least square (OLS), fixed effect model (FEM), and random effect model (REM). The Breusch Pagan (BP) and Lagrangian Multiplier (LM) tests formed the foremost step as this test can detect whether pooled or panel data is optimal. If the p-value of the BP test and the Chi-square of LM test is significant at 5% level, the panel data was chosen. Both FEM and REM were employed in this study to deal with panel data. The Hausman test was used to choose the suitable model for this research based on the null hypothesis. The FEM was chosen to analyse the data if the null hypothesis was rejected (or when the prob. < 0.05). Hence:

H0: The random effect is appropriate.

H1: the random effect is not appropriate.

The following empirical equations are proposed:

$$ef_{it} = \alpha_{0} + \alpha_{1} {\text{MaPP}}\_3_{it} + \mathop \sum \limits_{a = 1}^{5} \beta_{a} CC_{it} + \varepsilon_{it}$$

$$ef_{it} = \alpha_{0} + \alpha_{1} {\text{MaPP}}\_3_{it} + \alpha_{2} {\text{EPS}}_{it} + \alpha_{3} {\text{MaPP}}\_3_{it} {\text{*EPS}}_{it} + \mathop \sum \limits_{a = 1}^{5} \beta_{a} CC_{it} + \varepsilon_{it}$$

where ef_it_ = the log term of ecological footprint constant per capita of country i at time t. MaPP_3_it_ = macroprudential policies of country i at time t. EPS_it_ = environmental policy stringency of country i at time t. CC_it_ = control variable of country i at time t. ƹ_ijt_ = the error term.

## Empirical results

### Pre-empirical test

#### Descriptive statistics

Table 2 presents the descriptive statistics of variables of all countries in the sample.

**Table 2:** Descriptive statistics.

Variable	N	mean	sd	min	max
ef	319	1.463	0.653	− 0.377	2.391
MaPP_3	319	3.790	5.496	0	26
fp	319	17.97	3.362	9.802	24.93
GDP	319	9.697	1.243	6.303	11.01
trade	319	46.02	18.01	15.64	110.6
fdi	319	2.110	1.911	− 1.164	12.73
fd	319	100.3	51.79	6.626	217.8

In terms of the dependent variable, the mean logarithmic form of EF is 1.463 with a standard deviation of 0.653 while the minimum and maximum value of this variable is − 0.377 and 2.391. The average value of macroprudential policies is 3.790 with a standard deviation of 5.496 while the minimum and maximum value of the independent variable is 0 and 26.

Table 3 presents Pearson’s correlation matrix which shows the correlation findings between variables and the VIF value. Concerning the dependent variable, ecological footprint is observed positively correlates between fiscal policy, economic growth, openness index, foreign direct investment, financial development, as well as environmental policy stringency. Ecological footprint, contrariwise, negatively relates to macroprudential policies. The highest correlation coefficient (0.888) is between ef and GDP, demonstrating that the regression estimation is not multicollinear. According to^33^, a correlation coefficient as high as 0.842 leads to the conclusion that multi-collinearity is not a significant problem in the models. The correlation coefficient is also over 0.8^51^. The VIF value in Table 3 indicates that there is no significant multicollinearity among the variables in the regression model provided the maximum VIF value is 4.

**Table 3:** Correlation matrix and VIF.

	VIF	ef	MaPP 3	fp	GDP	trade	fdi	fd	EPS
ef	–	1							
MaPP_3	1.23	− 0.228***	1						
fp	2.27	0.573***	− 0.141**	1					
GDP	4	0.888***	− 0.246***	0.631***	1				
trade	1.76	0.270***	0.114**	0.386***	0.266***	1			
fdi	1.2	0.012	0.094*	0.047	− 0.012	0.213***	1		
fd	2.78	0.434***	− 0.061	0.045	0.573***	− 0.200***	− 0.023	1	
EPS	1.97	0.231***	0.118**	0.343***	0.529***	0.312***	− 0.174***	0.450***	

#### Cross-sectional dependence test

This study takes the Pesaran CD, Pesaran scaled LM, and Breusch-Pagan LM to test the cross-sectional dependence of data^52,52,53^. The null hypothesis is no cross-sectional dependence exists. In the results of tests shown in Table 4. One can see that three tests have p-values > 5%, which rejects the null hypothesis. These findings indicate that cross-sectional dependence be taken into account in the subsequent empirical estimation.

**Table 4:** Results of cross-sectional dependence tests.

Test	Statistics
Pesaran CD test	5.307***
Pesaran scaled LM test	5.327***
Breusch-Pagan LM test	2022.55***

Note: Significance level is denoted by *** for 1%, ** for 5% and * for 10%.

#### Unit root analysis

This study use^54^ CIPS test for the unit root analysis. The stationarity results are presented in Table 5. ef, MaPP 3, fp, GDP, trade, fd, and EPS showed significance at first difference while fdi are statistically significant at 1% level. The significance of the results rejected the null hypothesis of no stationarity.

**Table 5:** Unit root analysis.

Variable	I(0)	I(1)	Level of integration
ef	− 2.346	− 4.923***	I (1)
MaPP_3	− 1.135	− 3.950***	I (1)
fp	− 2.166	− 3.989***	I (1)
GDP	− 2.231	− 2.715*	I (1)
trade	− 2.496	− 3.961***	I (1)
fdi	− 3.333***	–	I (0)
fd	− 1.991	− 3.483***	I (1)
EPS	− 2.305	− 5.497***	I (1)

Note: Significance level is denoted by *** for 1%, ** for 5% and * for 10%.

### Basic results

In Table 6, this study includes MaPP_3 (macroprudential policies) and control variables in the model. In the preliminary stage, the results from Table 6 show that the fixed effect model is most suitable to be used in this study because the p value of BP test and Chi-square of LM test is significant at the 1% level or lower and the p value of Hausman test is significant at the 1% level or lower.

**Table 6:** Basic regression result.

	ols	fe	re
MaPP_3	0.000	− 0.011***	− 0.011***
	(0.00)	(0.00)	(0.00)
fp	− 0.010	− 0.032***	− 0.031***
	(0.01)	(0.00)	(0.00)
GDP	0.528***	0.563***	0.559***
	(0.03)	(0.03)	(0.03)
trade	− 0.000	− 0.003***	− 0.003***
	(0.00)	(0.00)	(0.00)
fdi	0.009	− 0.002	− 0.002
	(0.01)	(0.00)	(0.00)
fd	− 0.002***	− 0.001***	− 0.001***
	(0.00)	(0.00)	(0.00)
constant	− 3.306***	− 3.087***	− 3.067***
	(0.15)	(0.25)	(0.24)
N	319	319	319
r2	0.799	0.615	
r2_a	0.796	0.594	
F	207.240***	80.351***	
BP LM	chibar2(01) = 3183.49***		
chi2			534.834***
Hausman		chi2(6) = 17.94***	

The fixed effect model in Table 6 shows that macroprudential policies has a significant and negative effect on ecological footprint. A 1% increase in macroprudential policies leads to a decrease in ecological footprint by 0.011%. Regression results indicate that macroprudential policies negatively impact environmental deregulation in sample countries. Macroprudential policies function by influencing the actions of the financial sector and investment decisions. On the one hand, macroprudential policies have made institutions more inclined to lend to green practices, which can be effective in reducing EF. In addition, they have the potential to limit credit expansion^55^ and limited credit is accompanied by lower borrowing activities of families and enterprises^56^. Family and business activity reductions have a decisive role in improving energy efficiency and environmental quality. For instance, factories may reduce production, which would reduce energy use and reduce ecological footprint. Additionally, macroprudential instruments can distribute credit that is beneficial for eco-friendly sectors. On the other hand, macroprudential policies can encourage investment in green technology and eco-friendly initiatives. Loan-to-value ratios that are favorable to borrowers engaging in environmentally friendly activities make low-carbon investments more appealing^57^.

Regarding the control variables, regression results show that fiscal policy, trade, foreign direct investment, and financial development negatively contribute to the ecological footprint, while economic growth has a positive impact on the ecological footprint.

First, the results illustrate that, in the case of the top-11 largest economies, fiscal policy statistically increases environmental degradation. Fiscal policy can incentivize or provide subsidies for environmentally friendly activities^58^. These activities, such as the production of renewable energy or the use of energy-saving devices, can significantly contribute to reducing negative environmental impacts. Findings contradict the works of^59^ for Pakistan^60^, for China.

Second, results suggest the coefficient between EF and trade is significant and negative. This indicates that there is a technique effect, as described by^61^, wherein an expansion of trade is accompanied by more eco-friendly production practices due to advancements in technology. Results imply that trade openness will accelerate environmental sustainability. Findings are in line with the works of^62^ who selected Pakistan as research objective^63^, who selected a sample covering BRICS countries, and^64^ selected a sample covering 24 Organisation for Economic Co-operation.

Third, a statistically significant and negative relationship was found between financial development and EF. A 1% increase in financial development contributes to a 0.001% decrease in ecological footprint. Empirical results indicate that can financial development reduce negative environmental impacts. Energy is a primary determinant of environmental quality. Financial development can influence energy consumption by affecting environmental regulations^48^. The findings coincide with the findings of several studies, including^48^ for the top 10 pollutant footprint countries, and^65^ for APEC countries. Finally, foreign direct investment is insignificantly correlated with environmental sustainability. Similar results are found in the studies, including^66^ for South Africa, and^67^ for selected ASEAN countries.

Finally, regarding the role of GDP, regression results show that rapid economic growth may have negative effects on the environment. The composition effect is to blame for this phenomenon^68^. As the economy expands, so does the consumption of non-renewable resources, and industrialization is followed by an increase in negative environmental impacts.

### Quantile regression

In this step quantile regression is performed, which provides a more thorough analysis for model estimate at multiple quantiles, to obtain a more reliable conclusion^69^. This method not only provides results at different quantiles and complies with the non-normality requirements, but also solves issues with variable slope coefficient and cross-sectional dependence^70^. This study examines the impact of macroprudential policies on the ecological footprint at the 10th–90th quantiles. The results presented in Table 7 provide information on how the connection between variables may fluctuate in various distributions of ecological footprint. Macroprudential policies primarily reduce the ecological footprint in the first quantile (10%), but the effect diminishes until the fourth quantile (40%). However, the impact reverses in the medium quantile (50%) and shows a lower impact in the later quantiles (80%). The diminishing negative impact can be explained by different regulatory frameworks. Countries with lower ecological impact may already have stronger environmental rules. This provides a solid foundation for the effective implementation of macroprudential policies aimed at environmental protection. The positive effect of macroprudential policies on sustainability ends in the medium quantile and the negative impact diminishes in the later quantiles. This phenomenon can be referred as the “green paradox” since the response of the society is negative. The reversed effect can be attributed to a different level of trade-offs. In the medium quantile, countries may place a greater emphasis on economic development while environmental degradation is not serious. As a result, the effects of macroprudential policies are overshadowed by the drive for economic growth, leading to a positive effect. However, as environmental quality declines, public opinion and international pressure compel governments to strike a balance between economic and environmental concerns. This leads to a weakening of the positive effects of policies for economic growth that will increase negative environmental impacts.

**Table 7:** Quantile regression.

	Q10	Q20	Q30	Q40	Q50	Q60	Q70	Q80	Q90
MaPP_3	− 0.014***	− 0.011***	− 0.007***	− 0.004	0.009**	0.004	0.001	0.000	-0.000
	(0.00)	(0.00)	(0.00)	(0.00)	(0.00)	(0.01)	(0.01)	(0.01)	(0.00)
fp	0.008	− 0.000	− 0.002	− 0.002	0.002	− 0.003	− 0.042***	− 0.055***	− 0.051***
	(0.01)	(0.01)	(0.01)	(0.01)	(0.01)	(0.01)	(0.02)	(0.01)	(0.01)
GDP	0.511***	0.540***	0.546***	0.541***	0.516***	0.487***	0.540***	0.595***	0.624***
	(0.03)	(0.02)	(0.02)	(0.03)	(0.03)	(0.05)	(0.06)	(0.04)	(0.03)
fd	− 0.002***	− 0.001***	− 0.001***	− 0.001	− 0.000	0.000	− 0.002**	− 0.005***	− 0.006***
	(0.00)	(0.00)	(0.00)	(0.00)	(0.00)	(0.00)	(0.00)	(0.00)	(0.00)
fdi	0.017*	0.017***	0.014*	0.025**	0.029***	0.036**	− 0.004	− 0.011	− 0.013
	(0.01)	(0.01)	(0.01)	(0.01)	(0.01)	(0.02)	(0.02)	(0.01)	(0.01)
trade	− 0.004***	− 0.003***	− 0.004***	− 0.003**	− 0.002	0.001	0.005**	0.002	− 0.001
	(0.00)	(0.00)	(0.00)	(0.00)	(0.00)	(0.00)	(0.00)	(0.00)	(0.00)
constant	− 3.581***	− 3.693***	− 3.712***	− 3.702***	− 3.602***	− 3.329***	− 2.840***	− 2.619***	− 2.673***
	(0.17)	(0.11)	(0.13)	(0.17)	(0.18)	(0.28)	(0.32)	(0.24)	(0.17)
N	319	319	319	319	319	319	319	319	319

Standard errors in parentheses.

*p < 0.1, **p < 0.05, ***p < 0.01.

### Moderating effect of EPS

The varying levels of EPS may lead to differing degrees of cost associated with environmental pollution. To examine the moderating effect of EPS, this study includes the interaction term (MaPP_3* EPS) and EPS in the regression model. In line with basic regression findings, the coefficient of MaPP_3 is significantly negative at the 1% level. Furthermore, the interaction term (MaPP_3* EPS) exhibits a significant negative coefficient, as demonstrated in Table 8. This signifies that in situations of high EPS levels, the role of macroprudential policies in combating environmental degradation is strengthened. This can be explained by influencing the actions of financial institutions and enhanced impact of macroprudential tools. On the one hand, macroprudential policies can limit credit expansion and direct investment decisions. Meanwhile, a more stringent environmental regulatory environment also prompts financial institutions to consider whether the returns from investing in a particular company can be sustained at the expected level. On the other hand, the impact of macroprudential policies can be enhanced in a high EPS environment. For instance, where there is already a strong societal and governmental movement toward environmental protection, policies that encourage green investments or discourage environmentally harmful practices might have a more noticeable influence.

**Table 8:** Moderating effect.

	ols	fe	re
MaPP_3	0.012**	− 0.004**	− 0.003*
	(0.00)	(0.00)	(0.00)
EPS	− 0.222***	− 0.078***	− 0.079***
	(0.02)	(0.01)	(0.01)
Interaction	− 0.001	− 0.002**	− 0.002**
	(0.00)	(0.00)	(0.00)
fp	0.002	− 0.013***	− 0.013***
	(0.01)	(0.00)	(0.00)
GDP	0.559***	0.606***	0.589***
	(0.02)	(0.02)	(0.02)
trade	0.005***	− 0.001*	− 0.001
	(0.00)	(0.00)	(0.00)
fdi	− 0.028***	− 0.004	− 0.004
	(0.01)	(0.00)	(0.00)
fd	0.001	− 0.000	− 0.000
	(0.00)	(0.00)	(0.00)
constant	− 3.814***	− 3.926***	− 3.780***
	(0.12)	(0.23)	(0.21)
N	319	319	319
r2	0.888	0.735	
r2_a	0.886	0.719	
F	308.782***	103.848***	
BP LM	chibar2(01) = 2027.03***		
chi2			903.867***
Hausman		chi2(8) = 23.47***	

Standard errors in parentheses.

*p < 0.1, **p < 0.05, ***p < 0.01.

### Robust test

To confirm key findings, four robustness tests are run in this section. In order to explore the potential impact of external events on the nexus, this study first divided our sample into pre- and post-global financial crisis (GFC). To test the reliability of the fundamental regression model, this study then replaces the dependent variable and the independent variable. Additional control variables are also incorporated to revisit the underlying relationship.

#### Robust test1: pre-and post-GFC periods

This study investigates the relationship between MaPP_3 and ecological footprint in both the pre-and post-GFC eras for the purpose of getting a comprehensive result. This study looks into whether the nexus will be impacted by exogenous shocks. According to the research of^50,71^, this sample is divided into two time periods: before and after 2007, assuming that 2007 marked the beginning of the GFC. The years prior to 2007 are known as the pre-GFC period, while the years starting in 2008 are known as the post-GFC period.

As indicated in Table 9, the fixed effect model is most suitable for these results. MaPP_3 is negatively correlated with sustainable development during the post-GFC era. Intriguingly, MaPP_3 shows a positive substantial influence on environmental degradation in the time before the GFC. The emphasis on economic expansion and less-developed green technologies during the pre-GFC period may be responsible for the positive link. Economic growth was prioritized heavily during the pre-GFC period^72^. The emphasize on economic expansion may have led to the implementation of numerous policies^73^ that lessened or even eliminated the effects of MaPP_3, which could accelerate environmental degradation, even in the presence of MaPP_3. Additionally, there were few green technologies that were accessible and affordable in the pre-GFC period. The impact of MaPP_3 is therefore limited by technology constraints even if it can combat environmental degradation by guiding investment decisions and controlling credit. As a result, this estimation indicates that MaPP_3 deepens the negative environmental impacts. After the GFC, there may have been a boost in environmental awareness and technological developments that made MaPP_3 more effective. To gain an advantage in the next round of global competitiveness following the GFC period, several countries have chosen technological innovation as the top industrial development plan, such as Germany's Industries 4.0^74^.

**Table 9:** Robust test1: GFC.

	Pre-GFC			post-GFC		
	ols	fe	re	ols	fe	re
MaPP_3	0.004	0.007***	0.005**	0.018***	− 0.011***	− 0.011***
	(0.01)	(0.00)	(0.00)	(0.00)	(0.00)	(0.00)
fp	− 0.004	− 0.003	− 0.003	− 0.006	− 0.021***	− 0.010
	(0.01)	(0.00)	(0.00)	(0.01)	(0.01)	(0.01)
GDP	0.503***	0.294***	0.337***	0.589***	0.104	0.284***
	(0.03)	(0.04)	(0.03)	(0.05)	(0.06)	(0.05)
trade	0.005***	0.001	0.000	− 0.005***	-0.001	0.001
	(0.00)	(0.00)	(0.00)	(0.00)	(0.00)	(0.00)
fdi	− 0.012	− 0.002	− 0.003	0.009	0.003	0.004
	(0.01)	(0.00)	(0.00)	(0.02)	(0.00)	(0.00)
fd	− 0.001	0.000	0.000	− 0.002***	0.001**	0.001
	(0.00)	(0.00)	(0.00)	(0.00)	(0.00)	(0.00)
constant	− 3.367***	− 1.322***	− 1.714***	− 3.900***	0.826	− 1.225**
	(0.17)	(0.32)	(0.30)	(0.31)	(0.69)	(0.48)
N	176	176	176	143	143	143
r2	0.866	0.626		0.769	0.442	
r2_a	0.861	0.589		0.759	0.371	
F	181.991***	44.418***		75.334***	16.607***	
BP LM	chibar2(01) = 1105.59***			chibar2(01) = 414.80***		
chi2			301.908***			117.818***
Hausman		chi2(6) = 13.11**			chi2(6) = 31.50***	

Standard errors in parentheses.

*p < 0.1, **p < 0.05, ***p < 0.01.

#### Robust test2: replace dependent variable

Dependent variable is replaced with CO2 emissions metric tons per capita (CE) from WDI and consumption-based carbon emissions (CCO2) from the global carbon atlas (www.globalcarbonatlas.org). In line with the study of^6,75–79^, this study chooses CE (log.) as one indicator of the quality of the environment. Following the method of^80^, this study choose CCO2 (log.) as the proxy of environmental sustainability. The metric for computing CCO2, accounting for trade effects, considers the influence of international trade. Results are reported in Table 10. Regression findings demonstrate that the fundamental regression is still reliable.

**Table 10:** Robust test2: replace dependent variable.

	ols	fe	re	ols	fe	re
	CCO2	CCO2	CCO2	CE	CE	CE
MaPP_3	0.034***	− 0.010***	− 0.007***	0.004	− 0.012***	− 0.011***
	(0.01)	(0.00)	(0.00)	(0.01)	(0.00)	(0.00)
fp	− 0.154***	− 0.018***	− 0.022***	− 0.068***	− 0.031***	− 0.033***
	(0.01)	(0.01)	(0.01)	(0.01)	(0.01)	(0.01)
GDP	0.017	0.915***	0.843***	0.592***	0.843***	0.807***
	(0.05)	(0.04)	(0.04)	(0.04)	(0.04)	(0.04)
trade	− 0.000	− 0.005***	− 0.005***	0.010***	− 0.004***	− 0.004***
	(0.00)	(0.00)	(0.00)	(0.00)	(0.00)	(0.00)
fdi	0.001	0.010**	0.010**	− 0.015	− 0.001	− 0.001
	(0.02)	(0.00)	(0.00)	(0.01)	(0.00)	(0.00)
fd	0.008***	− 0.001***	− 0.001***	0.001	− 0.003***	− 0.003***
	(0.00)	(0.00)	(0.00)	(0.00)	(0.00)	(0.00)
constant	8.654***	− 1.172***	− 0.439	− 3.198***	− 5.271***	− 4.918***
	(0.31)	(0.33)	(0.39)	(0.25)	(0.35)	(0.35)
N	319	319	319	319	319	319
r2	0.588	0.754		0.679	0.655	
r2_a	0.580	0.741		0.673	0.637	
F	74.067***	154.243***		110.107***	95.530***	
BP LM	chibar2(01) = 2209.15***			chibar2(01) = 2998.94***		
chi2			692.839***			566.370***
Hausman		chi2(6) = 68.22***			chi2(6) = 25.46***	

Standard errors in parentheses.

*p < 0.1, **p < 0.05, ***p < 0.01.

#### Robust test3: replace independent variable

A variable called map_r5 are created, utilizing a 5-year spanning window to aggregate the macroprudential tools, whereas fundamental regression model uses 3-year rolling data, in accordance with the earlier study of^46^. Regression findings reported in Table 11 demonstrate that the fundamental regression is still reliable.

**Table 11:** Robust test3: replace independent variable.

	ols	fe	re
map_r5	0.002	− 0.007***	− 0.007***
	(0.00)	(0.00)	(0.00)
fp	− 0.011	− 0.031***	− 0.030***
	(0.01)	(0.00)	(0.00)
GDP	0.546***	0.589***	0.581***
	(0.03)	(0.03)	(0.03)
trade	− 0.002	− 0.005***	− 0.005***
	(0.00)	(0.00)	(0.00)
fdi	0.013	0.002	0.002
	(0.01)	(0.00)	(0.00)
fd	− 0.002***	− 0.001***	− 0.001***
	(0.00)	(0.00)	(0.00)
constant	− 3.409***	− 3.297***	− 3.235***
	(0.17)	(0.30)	(0.28)
N	297	297	297
r2	0.797	0.600	
r2_a	0.793	0.577	
F	190.010***	70.036***	
BP LM	chibar2(01) = 2707.20***		
chi2			465.658***
Hausman		chi2(6) = 14.54**	

Standard errors in parentheses.

*p < 0.1, **p < 0.05, ***p < 0.01.

#### Robust test4: add extra control variable

The reliability of empirical findings is likely to decrease, and estimation bias may occur due to the absence of relevant variables. As a result, the model is extended by one variable and check to see if the key results change. The variable of ICT is included in this analysis in line with the conclusions of earlier studies^68,76,77,81^, which highlight the significance of ICT and CPI in influencing environmental degradation. ICT is characterized by mobile subscriptions per 100 people while CPI is proxied by the consumer price index from WDI. Subsequently, this study introduces these variables one by one into the regression model. The corresponding results are listed in Table 12. The results of basic regression analysis are supported by the regression results.

**Table 12:** Robust test4: adding control variable ICT or CPI.

	ols	fe	re	ols	fe	re
MaPP_3	0.009**	− 0.009***	− 0.008***	0.014***	− 0.008***	− 0.007***
	(0.00)	(0.00)	(0.00)	(0.00)	(0.00)	(0.00)
fp	− 0.007	− 0.012***	− 0.013***	− 0.011	− 0.029***	− 0.028***
	(0.01)	(0.00)	(0.00)	(0.01)	(0.00)	(0.00)
GDP	0.561***	0.650***	0.628***	0.564***	0.664***	0.622***
	(0.03)	(0.03)	(0.02)	(0.03)	(0.03)	(0.02)
trade	0.000	− 0.001**	− 0.001**	− 0.001	− 0.004***	− 0.003***
	(0.00)	(0.00)	(0.00)	(0.00)	(0.00)	(0.00)
fdi	0.008	0.007**	0.006**	0.011	0.003	0.003
	(0.01)	(0.00)	(0.00)	(0.01)	(0.00)	(0.00)
fd	− 0.002***	− 0.000	− 0.000	− 0.001***	− 0.001***	− 0.001***
	(0.00)	(0.00)	(0.00)	(0.00)	(0.00)	(0.00)
ICT	− 0.002***	− 0.002***	− 0.002***			
	(0.00)	(0.00)	(0.00)			
CPI				− 0.005***	− 0.002***	− 0.002***
				(0.00)	(0.00)	(0.00)
constant	− 3.591***	− 4.404***	− 4.170***	− 3.302***	− 3.986***	− 3.614***
	(0.16)	(0.25)	(0.24)	(0.14)	(0.24)	(0.21)
N	319	319	319	319	319	319
r2	0.816	0.716		0.827	0.700	
r2_a	0.812	0.700		0.823	0.683	
F	197.596***	108.251***		212.570***	100.095***	
BP LM	chibar2(01) = 3257.84***			chibar2(01) = 2793.84***		
chi2			787.121***			761.842***
Hausman		chi2(7) = 19.23***			chi2(6) = 45.48***	

Standard errors in parentheses.

*p < 0.1, **p < 0.05, ***p < 0.01.

## Conclusion and policy recommendations

### Conclusion

This paper examines the effects of macroprudential policies on ecological footprint from 1992 to 2020 among the top 11 largest countries. Empirical findings support the notion that macroprudential policy and environmental sustainability are positively connected. A non-parametric method is especially well-suited for robust check in this study due to the wide range of ecological footprint levels in the sample. Macroprudential policies primarily reduce the ecological footprint in the first quantile (10%), but the effect diminishes until the fourth quantile (40%). In the medium quantile (50%), the impact reverses and shows a lower effect, which further diminishes in the later quantiles (80%). The effects of macroprudential policies are amplified by EPS. Additionally, this study notices that the effects of macroprudential measures during the post-GFC period are consistent with the fundamental regression model after separating the sample period into pre- and post-GFC periods. Nevertheless, it is interesting to mention the positive sign of macroprudential policies in the pre-GFC period. Robust testing supports fundamental regression findings.

### Policy recommendations

This research has several implications for policy.

First, given the positive relationship between macroprudential policies and sustainable development, governments should make an effort to include environmental issues in their macroprudential policy framework. Establishing systems to coordinate environmental protection with economic growth and make sure that policies reinforce one another. Additionally, based on the results of quantile regression, it’s important to strike a balance between economic development and environmental concerns. This may involve reevaluating policies to ensure that economic growth does not come at the cost of environmental degradation. Therefore, it is recommended to customize various types of macroprudential policies to mitigate potential conflicts with growth. Furthermore, environmental change must be fueled by public involvement and awareness. The public should be informed about the value of sustainability and included in the processes by which environmental policy is decided. Second, macroprudential policies can be used in conjunction with EPS, particularly those that deal with emissions and resource consumption, to encourage more sustainable economic operations. This can be accomplished by rewarding or subsidizing ecologically friendly practices and technologies. Finally, promoting green investment and innovation is indispensable. Financial incentives, better financing conditions for environmentally friendly projects, and funding for research and development into sustainable technology can impose a significant impact on raising environmental quality.

### Limitation and future direction

The study only examines how macroprudential policies affect ecological footprint in the top 11 largest countries. Nevertheless, geopolitical risks^82^, democratic accountability^83^ and economic uncertainty^84^ can impose an influence on the dependent variable, which is the main weakness of our research. Additionally, the impact of macroprudential policies and money policies on other indicator of environmental quality such as load capacity factor^85–88^ can be a future direction.
